# Safeguarding against Ebola: Vaccines and therapeutics to be stockpiled for future outbreaks

**DOI:** 10.1371/journal.pntd.0006275

**Published:** 2018-04-05

**Authors:** Eric M. Espeland, Chia-Wei Tsai, Joseph Larsen, Gary L. Disbrow

**Affiliations:** Biomedical Advanced Research and Development Authority (BARDA), Office of the Assistant Secretary for Preparedness and Response (ASPR), US Department of Health and Human Services (HHS), Washington, DC, United States of America; Institute for Disease Modeling, UNITED STATES

The Ebola virus outbreak of 2014 to 2016 had severe and devastating consequences for the people of West Africa, with more than 28,000 cases and 11,000 deaths across Liberia, Sierra Leone, and Guinea [1]. This epidemic exposed inadequacies in the medical countermeasure preparedness of international governments and organizations that limited their ability to effectively address the spread of the Ebola virus. Although Ebola virus had been circulating in Africa for decades with periodic outbreaks [1], funding for filoviruses has been limited to preclinical evaluation and establishment of assays and reagents that were necessary to quickly evaluate vaccine and therapeutic candidates. The few medical countermeasures that existed were stalled in early stages of development, a consequence of several factors, including insufficient funding to advance candidates, an uncertain regulatory path for development, and constraints associated with Biosafety Level 4 containment suites for research using Ebola viruses [2, 3, 4]. As the West Africa Ebola epidemic grew in scale, governments, international organizations, nongovernment organizations, and industry scrambled to mount an effective response to contain the outbreak [5]. The United States government (USG) and others invested millions of dollars to accelerate the development of vaccine, therapeutic, and diagnostic candidates from early preclinical development into manufacturing scale-up and clinical trials [2]. Several of these clinical trials were conducted in West Africa, which offered the potential to have a direct impact on the ongoing outbreak and demonstrate clinical efficacy of the medical countermeasures. Ultimately, the early development of these medical countermeasures was led by collaborative efforts across multiple organizations and countries. For example, early development of both vaccines and therapeutics can be attributed to organizations such as the National Institutes of Allergy and Infectious Diseases (NIAID), the US Army Medical Research Institute of Infectious Diseases (USAMRIID), the Defense Threat Reduction Agency (DTRA), the Medical Countermeasure Systems-Joint Vaccine Acquisition Program (MCS-JVAP), and the Public Health Agency Canada (PHAC). During the international response to the outbreak, numerous organizations, institutions, and international governments contributed to the evaluation of these medical countermeasures in the field, including the Ministries of Health in Guinea and Liberia, the Ministry of Health and Sanitation in Sierra Leone, the World Health Organization (WHO), and the Wellcome Trust, United Kingdom, to name a few.

The Biomedical Advanced Research and Development Authority (BARDA), part of the US Department of Health and Human Services, is mandated to support advanced research and development (R&D) of medical countermeasures for chemical, biological, radiological, and nuclear (CBRN) agents—including Ebola—under the Pandemic and All-Hazards Preparedness Act (PAHPA) of 2006 [6] and its reauthorization (PAHPRA) in 2013 [7] and procurement through the Project Bioshield Act of 2004 [8]. BARDA employs a public–private partnership model, providing funding, programmatic, and regulatory technical support for the advanced development of promising medical countermeasures toward licensure. In response to the West Africa outbreak, BARDA supported the development of Ebola vaccines and therapeutics candidates, with emphasis on late-stage development activities and manufacturing current Good Manufacturing Practice (cGMP) products for use in clinical trials, if deemed appropriate. As a result, several lead therapeutic and vaccine candidates may be eligible for Food and Drug Administration (FDA) licensure in the near-term and, more importantly, will be available for use during future public heath emergencies caused by the Ebola virus. Recently, BARDA announced four awards under Project BioShield to support the remaining late-stage development activities necessary for FDA licensure and for procuring these vaccines and therapeutics for the Strategic National Stockpile [9]. Project BioShield funding will support any Phase IV clinical study commitment required by the FDA once these vaccine and therapeutics have been licensed. BARDA’s continued support for the advanced development and procurement of these medical countermeasures will provide the USG with a robust response capability for Ebola virus, either through naturally emerging outbreaks or use as a bioweapon.

## Vaccines

Prior to the 2014 Ebola outbreak, most data on Ebola vaccines had been derived from nonclinical efficacy studies in small animals or nonhuman primates; clinical evaluations were limited [10,11]. The response to the West Africa Ebola outbreak accelerated the clinical evaluation and development of several Ebola vaccine candidates. Two lead candidates funded by BARDA—Merck’s V920 (rVSVΔG-ZEBOV-GP) and Janssen Vaccines & Prevention B.V.’s Ad26-ZEBOV/MVA-BN-Filo prime-boost vaccine (which has also received funding from NIAID)—are nearing consideration for licensure. The V920 vaccine produces a rapid immune response that is sustained up to one year post vaccination [12]. Merck is pursuing FDA licensure through a traditional approval pathway that emphasizes clinical efficacy data generated from the ring vaccination study conducted in Guinea [13]. The Ad26-ZEBOV/MVA-BN-Filo prime-boost vaccine is safe and well tolerated, producing sustained immune responses up to one year post vaccination [14, 15]. Janssen Vaccines & Prevention B.V. is pursuing FDA licensure through an Animal Rule/Accelerated pathway that will require demonstration of clinical efficacy through the establishment of an immune correlate within a nonhuman primate animal model. Both the V920 and the Ad26-ZEBOV/MVA-BN-Filo prime-boost vaccine candidates have been, or are being, evaluated in multiple Phase I, II, and III clinical trials.

## Therapeutics

ZMapp, an investigational drug in development by Mapp Biopharmaceutical, is a cocktail composed of 3 chimeric, monoclonal antibodies (mAbs) that target the Ebola virus glycoprotein (EBOV-GP). The efficacy of ZMapp was assessed in the PREVAIL II Phase I/II clinical trial in Guinea, Liberia, Sierra Leone, and the US during this outbreak. Although the predetermined statistical thresholds for success were not met due to limited enrollment during the final months of the outbreak, a trend towards efficacy was evident [16]. ZMapp is now widely considered to be a component of standard of care. As such, it was part of the response to the March 2016 Ebola flare-up that originated in the Nzérékoré prefecture in Guinea [17] and spread to Liberia, and the Zmapp drug was also available for use in the May 2017 outbreak in the Democratic Republic of the Congo. Mapp Biopharmaceutical, BARDA, and the FDA have partnered to make ZMapp available in the US, Liberia, Sierra Leone, and Guinea under an expanded access protocol to ensure continued availability to patients with Ebola virus disease.

REGN-3470-3471-3479, a fully human 3-mAb cocktail developed by Regeneron during the outbreak, targets EBOV-GP and is currently being evaluated in a Phase I clinical study (www.clinicaltrials.gov/ct2/show/NCT02777151). BARDA has collaborated with Regeneron since 2015 and provided funding for nonclinical studies, manufacturing, and a Phase I study. It is expected that REGN-3470-3471-3479 will further bolster the USG’s capability to deploy immunotherapeutics in the event of a public health emergency.

## Conclusion

While WHO declared the end of the West Africa Ebola epidemic in June 2016 [18], the 2017 outbreak in the Democratic Republic of Congo is a reminder that the Ebola virus will remain a security health threat. This outbreak highlights the need for improvements in the way we incentivize industry and coordinate domestic and international responses to make the necessary vaccines, diagnostics, and therapeutics to effectively respond to emerging and neglected tropical disease threats and other biothreats for which there may not be a commercial market. As the USG’s advanced development organization for medical countermeasures, BARDA is positioned to contribute to larger global initiatives—such as WHO’s R&D blue print (http://www.who.int/blueprint/about/en/) and efforts by the Center for Epidemic Preparedness Innovations (CEPI)—that address emerging and neglected tropical diseases when outbreaks of international concern arise. Coordination between these organizations, as well as other international stakeholders, is critical to ensure that appropriate resources and expertise are brought to bear during future outbreaks. In order to rapidly respond to novel threats, an emphasis on platforms that are capable of rapidly screening, identifying, and manufacturing vaccine or therapeutic candidates is needed. To this end, BARDA continues to assess and evaluate potential platform technologies as part of its larger portfolio of products, including efforts to develop medical countermeasures against emerging infectious diseases such as Middle East Respiratory Syndrome (MERS) and Zika.

There are a number of challenges that must be overcome to ensure adequate preparedness for future Ebola outbreaks, including completing the remaining advanced development activities necessary for regulatory approval and subsequent stockpiling of these medical countermeasures for use during a public health emergency. BARDA remains committed to making available safe and effective, FDA-approved vaccines and therapeutics for Ebola public health emergencies. Despite the advancement of the aforementioned vaccines and therapeutics against Ebola, gaps remain in our overall preparedness posture against other filoviruses. As such, BARDA will be pursuing the development of vaccines and therapeutics against Sudan ebolavirus and Marburg virus to address this gap. While we acknowledge that much work remains to prepare for future filovirus outbreaks, the recently announced BARDA awards for vaccines and therapeutics against Ebola represent an important milestone in our preparedness and ongoing commitment to counter this health security threat.
